# *In Vitro* Cercaricidal and Schistosomicidal Activities of the Raffia Wine and Hydroethanolic Extracts of *Pedilanthus tithymaloides* Linn (Poit). Stem Barks

**DOI:** 10.1155/2022/2672150

**Published:** 2022-09-16

**Authors:** Emilienne Tienga Nkondo, Hermine Boukeng Jatsa, Nestor Gipwe Feussom, Mérimé Christian Kenfack, Ulrich Membe Femoe, Stephanie Tamdem Guetchueng, Theodora Kopa Kowa, Pierre Kamtchouing, Louis-Albert Tchuem Tchuente

**Affiliations:** ^1^Laboratory of Animal Physiology, Department of Animal Biology and Physiology, Faculty of Science, University of Yaoundé I, P.O. Box 812, Yaoundé, Cameroon; ^2^Centre for Schistosomiasis and Parasitology, P.O. Box 7244, Yaoundé, Cameroon; ^3^Centre for Research on Medicinal Plants and Traditional Medicine, Institute of Medical Research and Medicinal Plants Studies, P.O. Box 13033, Yaoundé, Cameroon; ^4^Laboratory of Biology and Ecology, Department of Animal Biology and Physiology, Faculty of Science, University of Yaoundé I, P.O. Box 812, Yaoundé, Cameroon

## Abstract

Schistosomiasis control remains a public health concern, and there is a need to evaluate new strategies for targeting larval and adult stages of the parasite. As *Pedilanthus tithymaloides* is empirically used to treat schistosomiasis, it becomes essential to know its effective action scientifically. This study assessed the cercaricidal and schistosomicidal activity of *P. tithymaloides* stem barks raffia wine extract (Rw*Pt*) and hydroethanolic extract (He*Pt*). Different concentrations of these extracts were tested against cercariae (31.25–1000 *μ*g/mL) and adult worms (62.5–2000 *μ*g/mL) of *Schistosoma mansoni*. Niclosamide-olamine 5% (1 *μ*g/mL) and praziquantel (10 *μ*g/mL) were used as pharmacological controls. Cercariae viability was determined every 30 min for 180 min, and adult worms' motor activity and viability after 24 and 48 h incubation. In addition, cytotoxicity and phytochemical analysis were performed. He*Pt* was lethal to cercariae and adult worms with LC_50_ of 73.91 *μ*g/mL after 60 min of incubation and 731.17 *μ*g/mL after 48 h of incubation, respectively. Furthermore, a significant reduction of 94.44% in motor activity was observed in surviving worms at the concentration of 2000 *μ*g/mL. Rw*Pt* was less effective on *S. mansoni* cercariae with an LC_50_ of 617.86 *μ*g/mL after 180 min and on adult worms with a mortality rate of 9.83% at 2000 *μ*g/mL for 48 h incubation. Both extracts showed a weak cytotoxicity profile with an IC_50_ of 983.50 *μ*g/mL for He*Pt* and more than 1000 *μ*g/mL for Rw*Pt*. The LC-MS analysis of He*Pt* allowed the detection of two annotated diterpenoids. Based on the selectivity index, the hydroethanolic extract of *P. tithymaloides* stem barks disclosed an intense cercaricidal activity and a moderate schistosomicidal effect with low cytotoxicity. These findings may support the potential use of *Pedilanthus tithymaloides* as a natural product or a source of natural-derived compounds for interrupting schistosomiasis transmission.

## 1. Introduction

Schistosomiasis is a neglected tropical disease caused by the blood-dwelling fluke *Schistosoma*. Human schistosomiasis is the second most devastating tropical disease after malaria and remains a major public health problem. The World Health Organization estimated that 236 million people in the tropics and subtropics required preventive treatment in 2019, and 90% resided in Africa [1]. Schistosomiasis is endemic in 78 countries, causing roughly 70 million disability-adjusted life years. *Schistosoma* infection is also associated with high morbidity in human populations worldwide, with about 280,000 annual deaths related to its parasitism and complications [2, 3]. *Schistosoma mansoni*, the most widespread species, causes a yearly infection of 54 million people, and 393 million are at risk of getting infected in sub-Saharan Africa [4]. Infection occurs when free-swimming larvae, cercariae, released by various snail intermediate hosts penetrate the human host's skin while in contact with contaminated freshwater [1–3].

There is no effective vaccine [5, 6], and the main strategy for schistosomiasis control relies on mass drug administration (MDA) of praziquantel (PZQ). Although PZQ is effective against adult forms of all schistosome species, it is inefficient against larval and juvenile worms and, therefore, unable to prevent reinfection and stop parasite transmission [5–8]. In addition, repeated rounds of mass drug administration (MDA) with PZQ have led to therapeutic failures in disease-endemic areas, raising the possibility of drug resistance [9]. Indeed, *Schistosoma* isolates with reduced susceptibility to PZQ have already been identified, leading to concerns regarding treatment-resistant parasites [10]. Hence, there is a rising need for new therapeutic alternatives. Therefore, searching for bioactive natural products against *Schistosoma* is essential for establishing future strategies to control schistosomiasis [5, 11, 12]. In addition to treating infected individuals, interrupting people's contact with the infectious larvae cercariae could help avoid infection [13, 14].

In this regard, several *in vitro* studies have already been performed to search for new active compounds from medicinal plants against different stages of *S. mansoni*, and promising results have been reported [15–23]. *Pedilanthus tithymaloides* (Linn) Poit., also known as *Euphorbia tithymaloides*, is a common succulent shrub native to tropical and subtropical America belonging to the family Euphorbiaceae. The plant is actively used in India, Brazil, and Madagascar. Literature reported a wide range of healing properties [24], including anti-inflammatory and antioxidant [25–27], wound healing activity [28], larvicidal [29], antimalarial and antituberculous [30], anthelmintic [31], antimicrobial [32], antiviral [34], and antidiabetic [35]. The plant is widespread as ornamental in Cameroon's Littoral, Centre, East, Adamawa, and North regions. Some communities of the Littoral region mix the stem bark of this plant with raffia wine, boil the mixture, and drink it to treat schistosomiasis. Even though its biological potential has been proved against several microorganisms, the biological activity of *P. tithymaloides* against the life stages of *S. mansoni* has not yet been assessed. This study was carried out to evaluate the *in vitro* activities of the raffia wine extract and the hydroethanolic extract of *P. tithymaloides* stem bark on cercariae and adult worms of *S. mansoni* and to establish the extracts' cytotoxicity and phytochemical profiles.

## 2. Materials and Methods

### 2.1. Preparation of Plant Extracts

*Pedilanthus tithymaloides* (Linn) Poit. was harvested in May 2015 in the locality of Loum in the Littoral region of Cameroon. Botanical identification of a plant sample was performed at the “National Herbarium” of Yaounde, Cameroon, and a voucher specimen number 25714/SRF/Cam was deposited.

After plant collection, the raffia wine extract of *P. tithymaloides* stem bark was prepared as described by the traditional healer. First, barks (1500 g) were removed from the stems, cleaned, and boiled in fresh raffia wine (5 L). Next, the solution was concentrated under reduced pressure using a rotary evaporator (BÜCHI B-480), frozen, and then lyophilized to give the wine extract (Rw*Pt*) with an extraction yield of 2.64% w/w. For hydroethanolic extraction, the stem bark of *P. tithymaloides* was dried and powdered. Afterward, the powder (100 g) was boiled in 20% ethanol (1 L) for 2 h using a reflux design. Next, the solution was concentrated under reduced pressure using a rotary evaporator (BÜCHI B-480), frozen, and then lyophilized to obtain the hydroethanolic extract of *P. tithymaloides* stem bark (He*Pt*) with an extraction yield of 11.18% w/w.

### 2.2. Ethics Statement

All experiments in this study followed the principles of laboratory animals' use and care of the “European Community” guidelines (EEC Directive 2010/63/EEC). The protocol for experimental design was approved by the “Animal Ethical Committee” of the Laboratory of Animal Physiology, Faculty of Sciences, University of Yaounde I, Cameroon.

### 2.3. Evaluation of the In Vitro Cercaricidal Activity of the Plant Extracts

Raffia wine and hydroethanolic extracts of *P. tithymaloides* stem bark were tested for cercaricidal activity following the procedure previously described by some authors [16, 17, 36, 37].

#### 2.3.1. Preparation of Cercarial Suspension

*S. mansoni* cercariae were obtained by photostimulation of experimentally infected *Biomphalaria pfeifferi* snails. Briefly, 20 to 25 cercariae-shed snails were pooled into a glass beaker containing 20 mL of distilled water and exposed to artificial light for 2 hours. Snails were removed from the beaker, and the cercarial suspension was used for the assays. An average of 20 freshly shed cercariae were counted in 100 *μ*L of the suspension and transferred into each well of a 24 microtiter well plate under an inverted microscope (Olympus C.K. 2).

#### 2.3.2. Cercaricidal Activity Assays

The raffia wine or hydroethanolic extract of *P. tithymaloides* stem bark was dissolved in distilled water, and a stock solution of 2000 *μ*g/mL was obtained. Then, concentrations of 31.25, 62.5, 125, 250, 500, and 1000 *μ*g/mL were prepared by serial dilutions of the stock solution. Finally, each concentration was added to each well containing 20 cercariae for a final volume of 1 mL. Distilled water was set up as a control, and niclosamide-olamine 5% (1 g/mL) (Jiangsu Aijin Agrochemical Co., Ltd, China) as a pharmacological control. Each concentration was assayed in quadruplicates, and the experiments were replicated. The cercariae's viability assessment was performed for 180 minutes at 30 minutes intervals using an inverted microscope (Olympus C.K. 2). Cercariae were considered dead when they stopped movement and sank down, and their tail was detached. The LC_50_ value of *P. tithymaloides* raffia wine or hydroethanolic extract on *S. mansoni* cercariae was determined at different time points by interpolation from the “restricted cubic spline curve” (nonlinear regression). The minimal lethal concentration (MLC) (minimum concentration needed to kill all cercariae) and the minimal effective concentration (MEC) (minimum concentration required to observe any change in viability or morphology of cercariae) were determined after 180 minutes.

### 2.4. Evaluation of the In Vitro Schistosomicidal Activity of the Plant Extracts

#### 2.4.1. Worms' Recovery and Culture

Balb/c mice weighing 20–25 g were infected with 130 cercariae of *S. mansoni* released from experimentally infected *B. pfeifferi* by the tail and leg immersion technique. After 49 days, adult worms were recovered from mice's mesenteric veins and liver by perfusion with a sterile saline solution (0.9% NaCl) [38]. Freshly harvested worms were washed three times in a Glasgow Minimum Essential Medium (GMEM) (Sigma, St Louis, USA) supplemented with an antibiotic-antimycotic solution (10,000 U/mL penicillin, 10,000 *μ*g/mL streptomycin, and 25 *μ*g/mL amphotericin B) (Atlanta Biologicals, Lawrenceville, USA) and gentamicin (40 *μ*g/mL).

The bioassay followed the standard operating procedures that recommended at least five female and five male worms per well [39]. Therefore, 10 adult worms of both sexes were transferred to each well of a 24-well culture plate with 1900 *μ*L of complete GMEM, pH 7.5 (GMEM with 20 mM of HEPES, 40 *μ*g/mL gentamicin, 50 *μ*g/mL penicillin, 50 *μ*g/mL streptomycin, 100 *μ*g/mL neomycin, 2 mM of L-glutamine, and 5% heat-inactivated fetal bovine serum). The plates were then incubated for 2 hours at 37°C in a humid atmosphere of 5% CO_2_ to allow acclimatization.

#### 2.4.2. Schistosomicidal Activity Assays

After acclimatization of worms, the raffia wine extract (Rw*Pt*) or the hydroethanolic extract (He*Pt*) of *P. tithymaloïdes* stem bark was dissolved in distilled water, passed through a 0.2 *μ*m sterile syringe filter, and added to each well for final concentrations of 62.5, 125, 250, 500, 1000, and 2000 *μ*g/mL in a final volume of 2 mL. Worms incubated in GMEM alone or praziquantel (10 *μ*g/mL) were used as negative or positive controls, respectively. Each concentration was tested in quadruplicates, and the experiments were repeated. Worms were incubated for 48 h at 37°C, 5% CO_2,_ and monitored every 24 h to evaluate their motor activity and viability under an inverted microscope (Olympus C.K. 2) [17, 18]. Viability was determined based on the standard operating procedures for screening schistosomicidal compounds described previously ((+ + +) indicates normal activity; (+ +) slight loss of movement with active tail, suckers, and gynecophoric canal membrane; (+) movement of tails and suckers alone; and (−) no movement) [38]. Worms showing no sign of motility for 1 minute, associated with worm deformities such as blackening, twisting, and contracting, were considered dead [40]. The median lethal concentration (LC_50_) was calculated using the Trimmed Spearman–Karber (TSK) method, version 1.5 software downloaded from the U.S. Environmental Protection Agency [41].

### 2.5. Determination of the Cytotoxicity Profile of *Pedilanthus Tithymaloides* Extracts

The cytotoxicity analysis of the raffia wine and hydroethanolic extracts of *P. tithymaloides* was assessed using a C57/L melanoma liver cell line (Hepa 1–6 ATCC CRL-1830) based on the WST-8 assay. Cells were cultured in a high-glucose Dulbecco's minimal essential medium (DMEM) with pyruvate and L-glutamine (Gibco, Life Technologies, USA), supplemented with 10% heat-inactivated fetal bovine serum (FBS) (Serana, Australia) and 1% penicillin/streptomycin (Gibco, Life Technologies, USA). The cells were incubated at 37°C and 5% CO_2_. Monolayer cultures reaching a confluence between 80 and 90% were detached using trypsin solution (Sigma-Aldrich, Germany) and calibrated with a cell counter (Fast Read 102). The calibrated cell suspension was seeded into 96-well plates at 1 × 104 cells per well and incubated overnight for cell adhesion. Afterward, the medium was replaced with a fresh one, and cells were exposed to different concentrations (15.625, 31.25, 62.50, 125, 250, 500, and 1000 *μ*g/mL) of the extracts for 24 hours. Cells incubated in DMEM alone served as controls. Each concentration was tested in duplicates, and the experiment was repeated three times. Cells viability was measured by their mitochondrial activity in reducing 2-(2-methoxy-4- nitrophenyl)-3-(4-nitrophenyl)-5-(2, 4- disulfophenyl)-2H-tetrazolium monosodium salt to formazan using the Cell Counting kit-8 (WST-8, Abcam, ab228554, U.K.), according to the manufacturer instructions. The absorbance was measured at 450 nm using a Dynex MRX TC II microplate reader (Dynex Technologies, USA). Results were expressed as the percentage of cell growth, and the concentration of each extract required to inhibit cell growth by 50% (IC_50_) was calculated [37, 42].

### 2.6. Determination of the Selectivity Index

The degree of selectivity of each extract was measured as the ratio between the IC_50_ obtained for the cell line and the LC_50_ for *S. mansoni* cercariae or adult worms [16].

Selectivity index = [IC_50_ of extract in cell line (*μ*g/mL)]/[LC50 of the same extract in *S. mansoni* cercariae (*μ*g/mL)].

### 2.7. Liquid Chromatography-Mass Spectrometry (LC-MS) Analysis

The LC-MS analysis was performed using an Agilent 1200 Series liquid chromatography system coupled to an Agilent accurate QTOF 6520 mass spectrometer (GenTech Scientific, USA). Compounds were separated at room temperature on a Zorbax SB-C18 (50 × 2.1 mm, 1.8 *μ*m; Agilent Technologies, USA). The sample (1 mg/mL) was prepared in an HPLC-grade ACN followed by filtration through a Millipore filter (Sigma-Aldrich Ltd, France) and the injection volume was 0.8 *μ*L. The mobile phase was constituted of acetonitrile (containing 0.05% formic acid) and water (containing 0.05% formic acid). The method was as follows: 98% of ACN for 0.1 min, then 0% ACN for 8 min, followed by 0% ACN for 12.5 min, and then 98% of ACN for 25 min. The flow rate was 0.5 mL/min, and UV detection was set at 210 nm. For mass spectra acquisition, an Agilent accurate QTOF 6520 mass spectrometer with an electrospray interface in the positive mode (ESI+), temperature 340°C, and an ion spray of 4000 V was used. Sample cone voltage was set at 120 V, and mass spectra were recorded in the range m/z 50–2000. Nitrogen was used as nebulizer gas, and the nebulizer pressure was set at 30 psi. Data acquisition and analysis were performed with MassHunter Workstation software (Version B.04.00). The annotated compounds were drawn using Chemdraw Professional 15.0 (PerkinElmer informatics Inc., USA).

### 2.8. Statistical Analysis

Data analysis was conducted using GraphPad Prism 8.02 software (San Diego, CA, USA). All data were expressed as mean ± SEM. Significant differences between mean values were determined by two-way analysis of variance (ANOVA) for cercaricidal activity and one-way analysis of variance (ANOVA) for schistosomicidal activity, followed by the Tukey multiple-comparison test. Values of *p* < 0.05 were considered statistically significant.

## 3. Results

### 3.1. *In Vitro* Cercaricidal Activity of *Pedilanthus tithymaloides* Extracts

**Figure 1:**
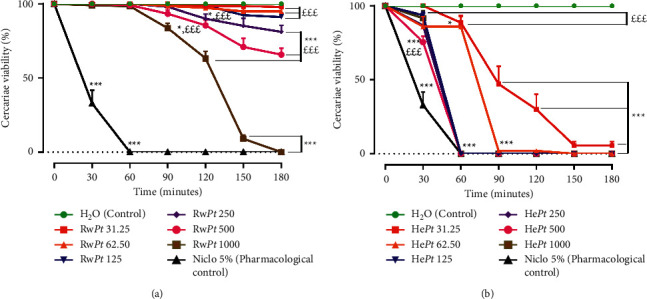
*In vitro* effect of the raffia wine (a) and the hydroethanolic (b) extracts of *Pedilanthus tithymaloides* stem bark on the viability of *Schistosoma mansoni* cercariae. Niclo 5%: niclosamide-olamine 5% (1 *μ*g/mL). Rw*Pt*: raffia wine extract of Pedilanthus tithymaloides stem bark (the number in front indicates the concentration of the extract). He*Pt*: hydroethanolic extract of Pedilanthus tithymaloides stem bark (the number in front indicates the concentration of the extract). Each point on the graph is expressed as mean ± SEM. ^*∗*^*p* < 0.05 and ^*∗∗∗*^*p* < 0.001: significantly different from the control (distilled water) at each time point. EEE*p* < 0.001: significantly different from the pharmacological control (niclosamide-olamine 5%) at each time point.

Different extracts of *Pedilanthus tithymaloides* stem bark showed varying cercaricidal potency against *S. mansoni* cercariae (Figure 1).

#### 3.1.1. Cercaricidal Activity of the Raffia Wine Extract of *Pedilanthus tithymaloides* Stem Bark

Effects of different concentrations of *P. tithymaloides* raffia wine extract (Rw*Pt*) on the viability of *S. mansoni* cercariae after incubation for up to 180 min are depicted in Figure 1(a). Following incubation with Rw*Pt*, a time- and dose-dependent decrease in the viability of cercariae was recorded. The reduction was insignificant for concentrations ranging from 31.25 to 125 *μ*g/mL. A significant decrease in cercariae viability was recorded only for concentrations from 250 to 1000 *μ*g/*μ*L. Still, it was less relevant than that of niclosamide-olamine 5% at the same time point (^*∗∗∗*^*p* < 0.001). Thus, cercariae incubated with 250 and 500 *μ*g/mL of Rw*Pt* showed the highest mortality rate of 18.93% (*p* < 0.001) and 34.27% (*p* < 0.001), respectively, at 180 min. At the highest concentration of 1000 *μ*g/mL, a slight reduction in cercariae viability appears within 60 min of incubation (16.45%), rising to 36.72% after 90 min, and became significant (*p* < 0.001) after 150 min (90.92%) and 180 min (100%). Cercariae remained viable for up to 180 min in the control group (Figure 1(a)). The minimal effective concentration (MEC) and the minimal lethal concentration (MLC) of the raffia wine extract of *P. tithymaloides* stem bark were 250 and 1000 *μ*g/mL, respectively, after 180 min of incubation (Table 1).

#### 3.1.2. Cercaricidal Activity of the Hydroethanolic Extract of *Pedilanthus Tithymaloides* Stem Bark

The exposure of cercariae to *P. tithymaloides* hydroethanolic extract (He*Pt*) for 30, 60, 90, 120, 150, and 180 min showed a time- and dose-dependent decrease in cercariae viability (Figure 1(b)). Within 60 min of incubation, all cercariae were dead at concentrations ranging from 125 to 1000 *μ*g/mL (*p* < 0.001), and there was no statistical difference with the reference drug niclosamide-olamine 5%. At 31.25 and 62.5 *μ*g/mL, a 14% cercariae mortality rate was recorded as compared to controls wells (*p* < 0.05). It was, however, less significant than the 100% mortality rate of niclosamide-olamine. After 150 min incubation, mortality rates of 93.60% and 100% were reached at 31.25 *μ*g/mL and 62.5 *μ*g/mL, respectively (*p* < 0.001). Cercariae remained viable for up to 180 min in the control group (Figure 1(b)). The minimal effective concentration (MEC) of the hydroethanolic extract of *P. tithymaloides* stem bark was 31.25 *μ*g/mL after 150 min of incubation, and the minimal lethal concentration (MLC) was 62.5 *μ*g/mL at the same time point (Table 1).

#### 3.1.3. Median Lethal Concentration of *Pedilanthus tithymaloides* Extracts

The median lethal concentration (LC_50_) of *P. tithymaloides* extracts was determined at each time point (Table 1). The LC_50_ of the raffia wine extract (Rw*Pt*) could only be calculated at 150 min and 180 min incubation. The LC_50_ values of the hydroethanolic extract (He*Pt*) decreased time dependently from 60 to 150 min of incubation. At 180 min time point, the LC_50_ of Rw*Pt* was 617.86 ± 34.65 *μ*g/mL, and that of He*Pt* was 19.26 ± 1.22 *μ*g/mL (Table 1). The hydroethanolic extract (He*Pt*) showed more pronounced cercaricidal potency than the raffia wine extract (Rw*Pt*).

### 3.2. *In Vitro* Schistosomicidal Effects of *Pedilanthus tithymaloides* Extracts

The schistosomicidal activity of different extracts of *P. tithymaloides* stem bark was assessed by microscopic observation of *S. mansoni* adult worms incubated at different concentrations (62.5–2000 *μ*g/mL) to check their motor activity and to determine the mortality rate using a viability scale. Results are shown in Table 2.

Control worms incubated only in the culture medium remained viable up to 48 h even though some displayed weak motor activity reduction. Incubation of adult *S. mansoni* worms with Rw*Pt* did not result in any worms' death at all concentrations after 24 h. Only a low mortality rate of 9.83% and 18.07% reduction of worms' motor activity were recorded at the highest concentration of 2000 *μ*g/mL after 48 h of incubation. The LC_50_ of Rw*Pt* was then greater than 2000 *μ*g/mL (Table 2). The minimal motor activity was marked by the absence of worm motility apart from gut movements, weak movement of the suckers, and occasional waves of the body.

Following exposure of adult worms to He*Pt*, the mortality rate increased in a time- and concentration-dependent manner. After 24 h of incubation, mortality rates of 10.98% and 25.60% were recorded at 1000 and 2000 *μ*g/mL, respectively. They were associated with reduced motility of surviving worms by 19.08% and 26.54%. After 48 h incubation, the mortality rates increased to 66.34% and 86.47% (*p* < 0.001) at 1000 and 2000 *μ*g/mL, respectively. At the same time, motor activity declined by 82.54% and 94.44%, respectively (Table 2).

The concentration of the hydroethanolic extract of *P. tithymaloides* stem bark necessary to kill 50% of worms (LC_50_) was 731.17 *μ*g/mL (365.74–1047.43 *μ*g/mL) after 48 h incubation. Although He*Pt* exhibited a lethal action on male and female worms, male worms seemed more sensitive than females. At the highest concentration of 2000 *μ*g/mL, 95.15% of male worms were killed against 78.33% of females (Table 2).

In the pharmacological control group incubated with 10 *μ*g/mL of praziquantel, parasite death was observed within 24 h post-incubation.

### 3.3. Effects of *Pedilanthus tithymaloides* Extracts on the Mouse Hepatoma Cells Proliferation

After 24 h incubation of mouse hepatic melanoma cells (Hepa 1–6 cells) with different concentrations (15.625 to 1000 *μ*g/mL) of raffia wine (Rw*Pt*) or hydroethanolic extract (He*Pt*) of *P. tithymaloides* stem bark, the inhibition rate on cell proliferation increased in a concentration-dependent manner. It reached 32.27% and 50.17% for Rw*Pt* and He*Pt*, respectively, at the highest 1000 *μ*g/mL concentration. Based on their inhibitory activity, the IC_50_ of Rw*Pt* was more than 1000 *μ*g/mL, and that of He*Pt* was 983.50 ± 7.75 *μ*g/mL (Table 3).

The selectivity index (SI) was calculated based on plant extracts' anthelmintic activity and cytotoxicity profile. The selectivity index of He*Pt* was 13.31 at 60 min and 51.06 at 150 min time points for the cercaricidal activity and 1.34 for the schistosomicidal activity. However, the IC_50_ of Rw*Pt* was more than 1000 *μ*g/mL, and its LC_50_ was more than 2000 *μ*g/mL for the schistosomicidal effect. Therefore, its selectivity index will be greater than 1.50 for cercaricidal activity and less than 1 for schistosomicidal activity (Table 3).

### 3.4. Phytochemical Analysis of the Hydroethanolic Extract of *Pedilanthus tithymaloides* Stem Bark

**Figure 2:**
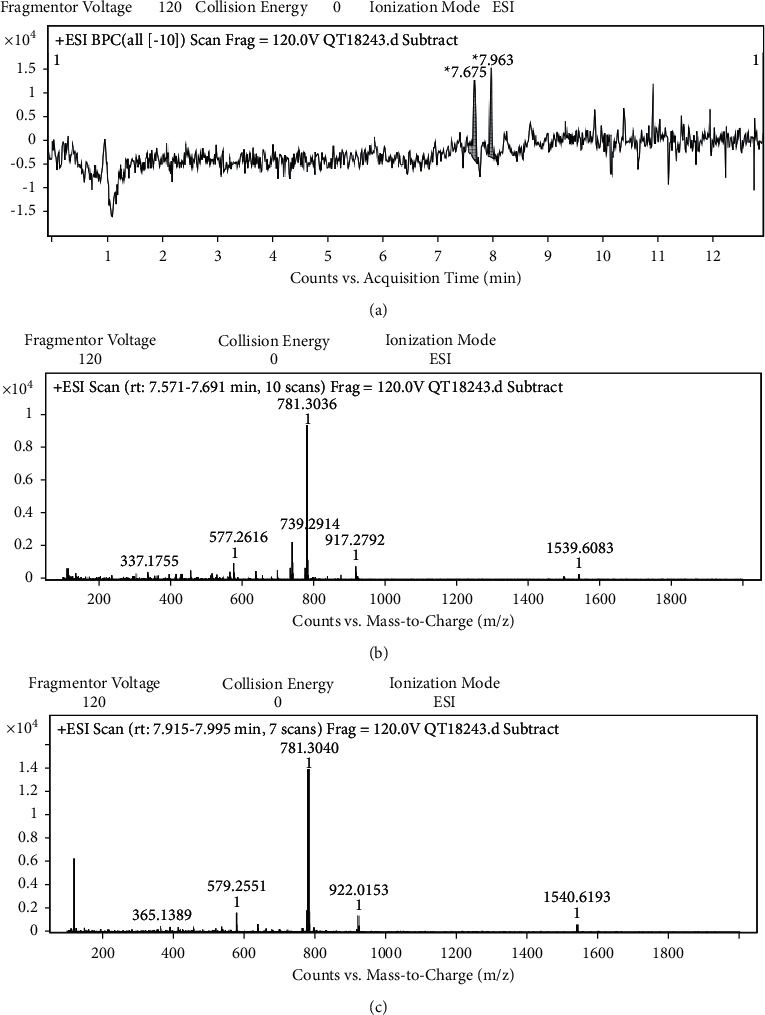
LC-MS chromatogram of the hydroethanolic extract of *Pedilanthus tithymaloides* stem bark (a) and spectra of annotated compounds (b and c).

**Figure 3:**
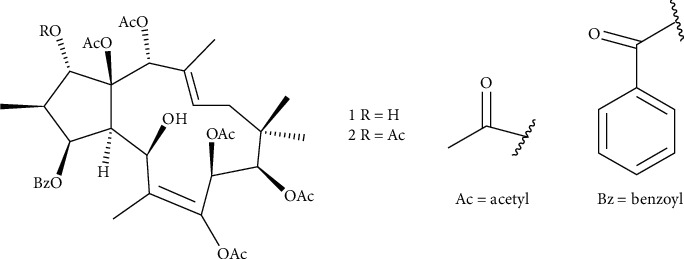
Chemical structures of annotated jatrophane diterpenoids detected in the hydroethanolic extract of *Pedilanthus tithymaloides* stem bark.

Analysis of the LC-MS chromatogram of the hydroethanolic extract from the stem bark of *P. tithymaloides* and comparison of the observed MS data with reported literature data on previously isolated compounds from the genus *Pedilanthus* and also with MS data of natural compounds listed in the Dictionary of Natural Products (https://dnp.chenetbase.com) revealed the possible presence of jatrophane diterpenes. The HPLC-MS showed two significant peaks with retention times (Rt) at 7.675 and 7.963 min (Figure 2(a)). The peak at Rt = 7.675 min corresponding to the molecular ion [M+Na]+ detected at m/z 739.2914 (Figure 2(b)) was annotated to the jatrophane diterpene 7, 8*β*, 9*β*, 14*α*,15*β*-pentaacetoxy-3*β*-benzoyloxy-1*α*, 5*β*-dihydroxyjatropha-6(7), 12-diene (1). The other peak at Rt = 7.963 min was annotated to a jatrophane diterpene, 1*α*, 7, 8*β*, 9*β*, 14*α*, 15*β*-hexaacetoxy-3*β*-benzoyloxy-5*β*-hydroxyjatropha-6(7), 12-diene (2) corresponding to the molecular ion [M+Na]+ detected at m/z 781.3040 (Figure 2(c)) (Table 4). Both compounds were previously reported from the methanolic extract of *P. tithymaloides* latex [26]. The chemical structures of these compounds are depicted in Figure 3.

## 4. Discussion

The search for new anti-*Schistosoma* drugs has intensified due to the report of some *S. mansoni* strains probably resistant to the reference drug, praziquantel. Consequently, there is an urgent need to discover cheap and safe alternatives for schistosomiasis control [5, 43].

Using medicinal plants as the best approach to identify new compounds to control schistosomiasis is one of the viable and promising research leads. Recently, several studies have demonstrated the potential effects of plant-derived products on cercariae and adult worms of *S. mansoni* [15–19, 21–23, 37]. Although the wide range of its pharmacological properties, the biological activities of *Pedilanthus tithymaloides* against the life stages of *S. mansoni* have not yet been reported. In this context, we have highlighted the antischistosomal activity of *P. tithymaloides* by *in vitro* tests using raffia wine and hydroethanolic extracts of the stem bark on *S. mansoni* cercariae and adult worms.

*In vitro* tests with different stages of *Schistosoma* are valuable tools to evaluate the schistosomicidal properties of a drug and determine its mode of action [44]. According to Moraes et al. [45], compounds with schistosomicidal activity can be effective in different ways: prophylactically by causing the death of cercariae and/or schistosomula, suppressively by inhibiting oviposition, and curatively by causing the death of adult worms. Therefore, we firstly examined the activity of the raffia wine and hydroethanolic extracts of *P. tithymaloides* against *S. mansoni* cercariae. Infection with schistosomes occurs when cercariae pass through unbroken skin and enter the body. As a result, interrupting the cercarial penetration into the skin is a potential mode of interrupting schistosomiasis transmission and thus the disease. Results of the current study showed varying cercaricidal activity of plant extracts. Although both extracts have inhibited cercariae viability, the hydroethanolic extract was the most potent by killing all cercariae over 60 minutes without any significant cytotoxicity against mouse hepatoma cells when tested at the same range of concentrations.

Our findings are comparable to the reported data on *Rauwolfia vomitoria* [16], *Ozoroa pulcherrima* [17], *Eryngium triquetrum* [46], *Balanites aegyptiaca* [47], *Caralluma dalzielli* [48], *Sida acuta,* and *Sida rhombifolia* [37]. However, at the same time of incubation (120 min), the hydroethanolic extract of *P. tithymaloides* was more potent than *R. vomitoria ethanolic* extract and *O. pulcherrima* aqueous extract. On the other hand, it was less active than *O. pulcherrima* methanolic extract and *S. acuta* and *S. rhombifolia* hydroethanolic extracts. Furthermore, researchers correlated the cercaricidal activity of plant extracts with their secondary metabolites. Various bioactive constituents such as steroids, alkaloids, flavonoids, tannins, phenols, diterpenes, triterpenes, coumarins, and saponins have already been identified in *Pedilanthus* sp. [24, 49, 50]. Therefore, although other possible active compounds might be present in the extract, we could link the cercaricidal activity of *P. tithymaloides* to the annotated diterpenoids 1 and 2. It has also been reported that the efficacy of *Origanum compactum* against *S. haematobium* cercariae was attributed to its terpenoids [51]. Most recently, Mohammed et al. [48] have assigned the anthelmintic activity of the aqueous extract of *Caralluma dalzielli* against the cercariae stage of *S. mansoni* to his high terpenoids content. On the other hand, Raveen et al. [29] have stipulated that the larvicidal action of *P. tithymaloides* leaves against the vector of the dengue fever *Aedes aegypti* could be assigned to flavonoids, phenols, and steroids that induced larval mortality. Moreover, the latex from *Euphorbia* sp. has been shown to act on both the snail vector and the parasite [46, 52]. Consequently, we could ascribe the cercaricidal activity of the hydroethanolic extract of *P. tithymaloides* stem bark to its terpenoids, phenols, flavonoids, and steroids contents.

The ability of *P. tithymaloides* to kill both adult male and female schistosomes was also evaluated during this study. Our results showed that the hydroethanolic extract of *P. tithymaloides* stem bark induced worms' death associated with the significantly reduced motor activity of surviving ones. Previous assays screening the schistosomicidal activity of some plant extracts or isolated compounds have revealed similar results [13, 14, 16–18, 21, 22, 35, 53–56]. However, the difference in survival time between male and female worms suggested that males were more susceptible than females. Similar findings were reported by others while exposing schistosomes to medicinal plant-derived compounds or products such as Garcinielliptone FC [57], dihydrocitronellol [58], an alkaloid-rich fraction from *Ruta graveolens* L. [56], and the Brazilian red propolis [19]. The schistosomicidal activity of the hydroethanolic extract of *P. tithymaloides* stem bark may be due to its jatrophane diterpenoids. Such compounds have been previously identified in the methanolic extract of *P. tithymaloides* latex [30]. Furthermore, they have been reported for their antimalarial and antituberculous activities and antiplasmodial activity against the K1 strain of *Plasmodium falciparum* [30, 58]. Additionally, some terpenoids are known to kill adult *S. mansoni* worms by altering their cholinergic nervous system [59]. The motility of schistosomes implies neurotransmitters such as acetylcholine, serotonin, neuropeptides, and glutamate [60–62]. The reduction in *S. mansoni* motor activity after incubation in the hydroethanolic extract of *P. tithymaloides* stem bark could be the consequence of its interference with the mechanism of concentration-relaxation of worm's smooth muscles [5]. It has also been demonstrated that terpenoids cause complete separation of paired worms and tegumental disruption [63]. Through this action, they can impair the parasite's motility. Therefore, the effectiveness of the hydroethanolic extract of *P. tithymaloides* stem bark against *S. mansoni* worms may be due, at least in part, to the jatrophane diterpenoids.

Establishing that a drug candidate cannot induce cytotoxicity at therapeutic concentrations is crucial. The US NCI plant screening program stipulates that a crude extract with *in vitro* cytotoxic activity has an IC_50_ less than 20 *μ*g/mL following incubation of cells between 48 h and 72 h [64]. According to this, IC_50_ of the hydroethanolic extract (983.50 *μ*g/mL) was approximately 50 times greater than 20 *μ*g/mL, indicating the very low cytotoxicity of this extract on mouse hepatoma cell lines. The relative effectiveness of a drug candidate in inhibiting parasite growth compared to inducing cell death is defined as the therapeutic index or selectivity index. Therefore, it is desirable to have a high therapeutic index (>2), giving a maximum anthelmintic activity with minimal toxicity on the cells [16]. The selectivity index of the hydroethanolic extract of *P. tithymaloides* stem bark was 19.26 for cercaricidal activity and 1.34 for schistosomicidal activity. These findings reveal that this extract is strongly active against *S. mansoni* cercariae but relatively effective against adult schistosomes.

## 5. Conclusions

The results of the current study provide evidence of the predominant cercaricidal activity and the moderate schistosomicidal activity of the hydroethanolic extract of *Pedilanthus tithymaloides* stem bark, with no cytotoxicity on mouse hepatoma cells. Also, we annotated two jatrophane diterpenes from this active extract. These findings may support the potential use of *Pedilanthus tithymaloides* as a natural product or a source of natural-derived compounds for interrupting schistosomiasis transmission. However, further investigations are in progress to disclose the pharmacological effects of these plant extracts through *in vivo* models of *Schistosoma mansoni* infection.

## Figures and Tables

**Table 1:** Effective and lethal concentrations of the raffia wine extract and the hydroethanolic extract of *Pedilanthus tithymaloides* stem bark against *Schistosoma mansoni* cercariae.

	Time (min)	Rw*Pt*	He*Pt*
LC_50_ (*μ*g/mL)	30	—	—
	60	—	73.91 ± 3.31 CI (51.84–82.70)
	90	—	34.07 ± 3.91 CI (18.15–45.46)
	120	—	28.84 ± 3.30 CI (18.15–43.41)
	150	665.14 ± 42.41 CI (454.36–778.57)	19.26 ± 1.22 CI (13.70–25.73)
	180	617.86 ± 34.65 CI (456.33–732.30)	19.26 ± 1.22 CI (13.70–25.73)
MEC (*μ*g/mL)		250	31.25
MLC (*μ*g/mL)		1000	62.50

Rw*Pt*: Raffia wine extract of *Pedilanthus tithymaloides* stem bark. He*Pt*: hydroethanolic extract of *Pedilanthus tithymaloides* stem bark. LC_50_: median lethal concentration. MLC: minimum lethal concentration. MEC: minimum effective concentration. CI: confidence interval.

**Table 2:** *In vitro* schistosomicidal activities of *Pedilanthus tithymaloides* raffia wine and hydroethanolic extracts after 24 and 48 hours of incubation.

Groups	Concentrations (*μ*g/mL)	Mortality rate (%)^a^		Dead females (%)^b^	Dead males (%)^c^	Motor activity reduction (%)^d^		Lethal concentration 50 (*μ*g/mL)
		Incubation period				Incubation period		
		24 h	48 h			24 h	48 h	
Control (GMEM)	—	0	0	0	0	0.69	6.96	—

Praziquantel	10	100	100	100	100	—	—	—

Rw*Pt*	2000	0	9.83	11.80	8.34	7.06	18.07	>**2000**
	1000	0	1.14	0	2.00	0	9.30	
	500	0	1.50	0	1.78	0	5.40	
	250	0	0	0	0	0	0	
	125	0	0	0	0	0	0	
	62.5	0	0	0	0	0	0	

He*Pt*	2000	25.60	86.47	78.33	95.15	26.54	94.44	**731.17** CI (365.74–1047.43)
	1000	10.98	66.34	52.03	81.46	19.08	82.54	
	500	8.94	24.39	6.06	39.74	11.92	35.97	
	250	6.99	26.63	0	34.80	9.45	30.31	
	125	1.63	5.07	0	10.84	4.86	12.28	
	62.5	3.22	13.68	8.29	18.48	0.74	12.17	

GMEM: Glasgow minimal essential medium. Rw*Pt*: raffia wine extract of *Pedilanthus tithymaloides* stem bark. He*Pt*: hydroethanolic extract of *Pedilanthus tithymaloides* stem bark. ^a^Percentages relative to all worms. ^b^Percentages relative to female worms after 48 h incubation. ^c^Percentages relative to male worms after 48 h incubation. ^d^Percentages relative to surviving worms. —: not applicable. CI: confidence interval.

**Table 3:** Cytotoxicity profile and selectivity index of *Pedilanthus tithymaloides* extracts for the cercaricidal and schistosomicidal activities.

Cytotoxicity				Cercaricidal activity		Schistosomicidal activity	
Extracts	Concentrations (*μ*g/mL)	Inhibition rates (%)	CI_50_ (*μ*g/mL)	LC_50_ (*μ*g/mL)	SI	LC_50_ (*μ*g/mL)	SI
Rw*Pt*	1000	32.27 ± 0.16	>**1000**	665.14 ± 42.41	>**1.50**	>2000	<**1**
	500	23.69 ± 0.03					
	250	18.67 ± 0.57					
	125	16.24 ± 1.01					
	62.5	15.67 ± 0.75					
	31.25	13.65 ± 0.15					
	15.625	9.03 ± 1.03					

He*Pt*	1000	50.17 ± 0.23	**983.50** **±** **7.75**	19.26 ± 1.22	**51.06**	731.17	**1.34**
	500	36.72 ± 3.51					
	250	37.10 ± 1.92					
	125	39.47 ± 0.28					
	62.5	37.35 ± 1.44					
	31.25	35.09 ± 1.36					
	15.625	27.63 ± 0.20					

SI: selectivity index. Rw*Pt*: raffia wine extract of *Pedilanthus tithymaloides* stem bark. He*Pt*: hydroethanolic extract of *Pedilanthus tithymaloides* stem bark.

**Table 4:** Main signals exhibited in the LC-MS spectra of compounds detected in the hydroethanolic extract of *Pedilanthus tithymaloides* stem bark and proposed attribution.

N°	Retention time (min)	m/z [M+Na]+	Molecular formula	Annotated compounds	Reference
1	**7.675**	739.2914	C37H48O14Na	7,8*β*,9*β*,14*α*,15*β*-pentaacetoxy-3*β*-benzoyloxy-1*α*,5*β*-dihydroxyjatropha-6(7),12-diene	[26]
2	**7.963**	781.3040	C39H50O15Na	1*α*,7,8*β*,9*β*,14*α*,15*β*-hexaacetoxy-3*β*-benzoyloxy-5*β*-hydroxyjatropha-6(7),12-diene	[26]

## Data Availability

The data used to support the findings of this study are available from the corresponding author upon request.
